# The Association between Maternal B Vitamins in Early Pregnancy and Gestational Diabetes Mellitus: A Prospective Cohort Study

**DOI:** 10.3390/nu14235016

**Published:** 2022-11-25

**Authors:** Na Wang, Tianchun Zhou, Xiaoxia Ma, Yuping Lin, Yan Ding

**Affiliations:** 1 Nursing Department, Obstetrics and Gynaecology Hospital of Fudan University, Shanghai 200090, China; 2 School of Nursing, Fudan University, Shanghai 200032, China

**Keywords:** B vitamins, vitamin B_1_, vitamin B_6_, vitamin B_12_, gestational diabetes mellitus

## Abstract

Background: This study evaluated the association between maternal B vitamins in early pregnancy and gestational diabetes mellitus (GDM) risk. Methods: A cohort of 1265 pregnant women was recruited at 8–15 weeks of gestation in 2021–2022 (Shanghai, China). Pregnancies with both serum B vitamin measurements at recruitment and glucose measurements at 24–28 weeks of gestation were included in the final analysis. Results: Of the 1065 pregnancies, in the final analysis, GDM occurred in 121 women (11.36%). In multivariate logistic models, an increased risk trend across serum vitamin B_1_ quartiles with GDM was observed (*p*-Trend = 0.001). Compared with women in the lowest quartile of serum vitamin B_6_, those in the upper two quartiles had approximately twofold higher odds of GDM. Moreover, compared with women with vitamin B_12_ levels < 150 pmol/L, those with vitamin B_12_ levels > 150 pmol/L had lower odds of GDM (*p* = 0.005). The restricted cubic spline regression models also revealed that serum vitamin B_6_ and vitamin B_12_ were associated with an increased risk of GDM in a nonlinear fashion. Conclusions: Our study shows that higher maternal serum vitamin B_1_ and B_6_ levels in early pregnancy are associated with increased GDM risk, while sufficient vitamin B_12_ status is associated with lower GDM risk.

## 1. Introduction

Gestational diabetes mellitus (GDM) is one of the most common metabolic disorders during pregnancy, affecting 16.7% of pregnancies worldwide [1]. The prevalence is 14.8% in China [2]. GDM is related to higher short-term and long-term adverse outcomes in both mothers and offspring [3,4,5]. In addition, adverse metabolic programming of offspring may exist prior to the diagnosis of GDM [6]. Thus, identifying modifiable risk factors for GDM would be useful for the prevention of this disease.

Balanced nutrition is important for pregnant women. During pregnancy, severe micronutrient deficiency or excess can have negative impacts on both the fetus (including low birth weight, intrauterine growth retardation or congenital malformations) and the pregnant women (hypertensive disorders or gestational diabetes) [7,8,9]. Group B vitamins, particularly thiamine (B_1_), riboflavin (B_2_), niacin (B_3_), pyridoxine (B_6_), folate and cobalamin (B_12_), have important roles in glucose metabolism and most have been linked to type 2 diabetes [10,11]. Folate and vitamin B_12_ are essential nutrients for the metabolism of the one-carbon unit involved in the DNA methylation and synthesis of amino acids, lipids and nucleic acids [12]. In order to prevent neural tube defects, supplementation of 400 µg folic acid daily is routinely recommended for women of childbearing age from at least 3 months before conception and during pregnancy [13,14].

Recently, folate and vitamin B_12_ have been studied regarding their relationship with GDM risk, but conflicting results have been reported [15,16]. In several studies, vitamin B_12_ insufficiency and folate excess were common in early pregnancy, and a higher serum folate/vitamin B_12_ ratio was associated with an elevated risk of GDM [16,17]. These findings highlight the detrimental effects of maternal imbalance of these two vitamins. Vitamin B_12_ is also a coenzyme involved in the degradation of odd-chain fatty acids and branched-chain amino acids (BCAAs) [18]. Elevated BCAA levels play a role in the onset of type 2 diabetes [19].

Today, increasing numbers of pregnant women are taking compound vitamin supplements mainly containing folic acid and other B-group vitamins. In fact, B vitamins are often metabolically entwined and some of the mechanisms of their roles could contribute to glucose homeostasis. For example, vitamin B_6_ is also involved in one-carbon and homocysteine metabolism, and it can promote the absorption of vitamin B_12_ [20]. Vitamin B_1_ is involved in many redox reactions in glucose and BCAA metabolism [21]. Vitamin B_1_ homeostasis disturbance is prevalent in type 1 and type 2 diabetes [21,22], but its role in glucose metabolism during pregnancy is unclear. Although several B vitamins (such as vitamin B_1_, vitamin B_2_ and vitamin B_6_) are included in dietary supplements, their individual metabolic roles are not well specified in pregnancy [23].

To our knowledge, limited studies have investigated the relationship between B vitamins other than folate and vitamin B_12_ in early pregnancy and GDM. Therefore, the aims of this prospective cohort study were to (1) describe the serum levels of B vitamins including folate and vitamins B_1_, B_2_, B_6_ and B_12_ in early pregnancy; and (2) investigate whether serum B vitamins in early pregnancy are associated with glucose levels and the risk of GDM.

## 2. Materials and Methods

### 2.1. Study Population

A prospective cohort study to investigate the influences of maternal dietary supplements and nutritional biomarkers on blood glucose levels and GDM during pregnancy was conducted among pregnant women in a hospital in Shanghai, China. The Research Committee of the study hospital approved this study (No. 202123), and all participants gave written informed consent to participate. In brief, all participants were recruited from a maternity hospital, which is a tertiary university-affiliated hospital located in Shanghai. Annually, the total number of births is about 12,000 in the hospital. From March 2021 to March 2022, two research nurses enrolled women at their first antenatal visits. Participants were eligible for our study if they (1) had a live singleton pregnancy at 8–15 weeks’ gestation and (2) had registered and planned to give birth in the study hospital. The exclusion criteria were (1) pre-existing diabetes or a diagnosis of GDM before 24 weeks of gestation; (2) a previous pregnancy with a neural tube defect; (3) chronic viral hepatitis, cirrhosis or severe liver disease; and (4) multiple gestation. In total, 1800 pregnant women were approached and 1265 women were recruited after assessing their eligibility. We restricted our study sample to pregnant women with complete measurements of folate and vitamins B_1_, B_2_, B_6_ and B_12_ at 8–15 weeks of gestation and three glucose measurements by oral glucose tolerance test (OGTT) at 24–28 weeks of gestation. These inclusion criteria resulted in 1065 participants in the final analysis.

### 2.2. Data Collection

Baseline data were collected via face-to-face interviews using a self-reported questionnaire. The data collected included the following: demographics (age, educational background and monthly personal income), lifestyle characteristics (smoking, passive smoking, alcohol consumption and physical activity), supplement intake (brand, type and duration), and medical, reproductive and family history. The medical histories of the participants were cross-checked with the electronic medical records from the hospital. Weights were measured at recruitment and OGTT visits. We calculated weight gain by subtracting self-reported pre-pregnancy weight from the weight measured at each visit. Pre-conceptional body mass index (BMI) was calculated through self-reported pre-pregnancy weight and height and divided into four categories: underweight (BMI below 18.5 kg/m^2^), normal weight (BMI of 18.5 to 23.9 kg/m^2^), overweight (BMI of 24.0 to 27.9 kg/m^2^) or obese (BMI of 28.0 kg/m^2^ or higher); these are the BMI cutoffs for Chinese individuals [24]. Physical activity level was assessed according to the International Physical Activity Questionnaire-Short Form [25], from which metabolic equivalent (MET)-min/week was calculated. Smoking exposure was defined as smoking or passive smoking 3 months before or during pregnancy. Alcohol consumption was defined as drinking any alcoholic beverages 3 months before or during pregnancy. B vitamin supplementation was regarded as regularly taking folic acid or compound vitamin supplements 3 months before and during pregnancy. In general, the folic acid supplement contains 0.4 mg/pill of folic acid, and the compound vitamin supplements contain 0.4 mg/pill or 0.8 mg/pill of folic acid, combined with other B vitamins depending on the brands.

### 2.3. Diagnosis of GDM

In accordance with the criteria developed by the International Association of Diabetes and Pregnancy Study Groups, participants underwent a 75 g OGTT between 24 weeks and 28 weeks of gestation and GDM was diagnosed if any of the following criteria were met: fasting glucose ≥5.1 mmol/L, 1 h glucose ≥10.0 mmol/L, 2 h glucose ≥8.5 mmol/L, or any combination of these [26].

### 2.4. Biochemical Analysis

As part of routine antenatal care, blood samples were collected at recruitment and at 24–28 weeks of gestation from all participants by trained nurses. Samples were centrifuged at 3000 rpm for 5 min to separate serum or plasma for biochemical analysis. B vitamin levels were measured immediately on a vitamin analyzer (VSS-A-01, Chongqin, China) using electro-chemiluminescent assays (Synovie). Plasma glucose levels were measured by the electrochemical glucose oxidase method using an automatic biochemical analyzer (Hitachi 7600, Tokyo, Japan). All measurements were conducted by the professional staff in the biochemical laboratory of the study hospital. The inter-assay coefficients of variation were <10% for the entire measurements.

### 2.5. Statistical Analysis

R 4.2.1 and Stata 16.0 (Stata Corp., College Station, TX, United States) were used for all the analyses. Categorical variables were described as frequencies and percentages. Continuous data were summarized as means and standard deviations or medians and interquartile ranges. Comparisons between groups for categorical variables were performed using χ^2^ tests. Comparisons between groups for continuous variables with normal distribution were analyzed using analysis of variance (ANOVA) or unpaired Student t-tests, and continuous variables with skewed distributions were performed by Kruskal–Wallis tests. Vitamin B_12_ insufficiency was defined as <150 pmol/L, which is often used to define vitamin B_12_ deficiency in pregnant women [27]. Folate insufficiency was defined as <5.9 nmol/L, which is suggested to define folate deficiency in the first trimester of pregnancy [28]. Other serum B vitamins were only placed into quartiles because there are no specified cut-off values for pregnant women. Correlation analysis was performed to investigate the relationship among serum B vitamins, fasting, 1 h and 2 h plasma glucose. Multivariable logistic regression models were used to explore the associations of these serum B vitamins with GDM, with adjustment for age, education, parity, first-degree family history of diabetes, smoking exposure, alcohol consumption, pre-conceptional BMI, gestational weight gain at OGTT visit and physical activity levels. Odds ratios (OR) and 95% confidence intervals (CI) were reported. Moreover, restricted cubic spline (RCS) regression models with assumed three knots were used to outline the potential nonlinear relationships between continuous serum B vitamins and GDM risk. A two-tailed *p* value of <0.05 was regarded as statistically significant.

## 3. Results

### 3.1. Baseline Characteristics

Table 1 shows the demographic characteristics and the biochemical measurements of the study population, according to GDM status. Among 1065 pregnancies, GDM occurred in 121 women (11.36%). The mean (standard deviation) age was 30.8 (3.7) years. Of the 1065 participants, 89.1% were nulliparous, with more than 90% having a college or above degree. Overweight and obese women accounted for 16.9% while 14.9% of the women were found to be underweight. B-vitamin supplement intake was found in 94.3% of pregnant women, of which 68.4% took multivitamin supplements. The median (interquartile range) concentrations of serum vitamin B_1_, vitamin B_2_, vitamin B_6_, folate and vitamin B_12_ were 86.5 (75.1–98.5) pmol/L, 13.5 (12.3–14.9) pmol/L, 27.2 (24.2–35.7) pmol/L, 11.8 (10.1–13.9) nmol/L and 174.8 (132.6–210.3) pmol/L. Compared with women without GDM, women with GDM were more likely to be older (*p* < 0.001) and multiparous (*p* = 0.037), overweight or obese before pregnancy (*p* < 0.001), and have a higher first-degree family history of diabetes (*p* < 0.001). In addition, women with GDM had significantly higher levels of serum vitamin B_1_ (*p* < 0.001) and lower levels of vitamin B_12_ (*p* = 0.038), and more had vitamin B_12_ insufficiency (*p* = 0.003).

### 3.2. Correlations between Serum B Vitamins and Glucose Levels

**Figure 1:**
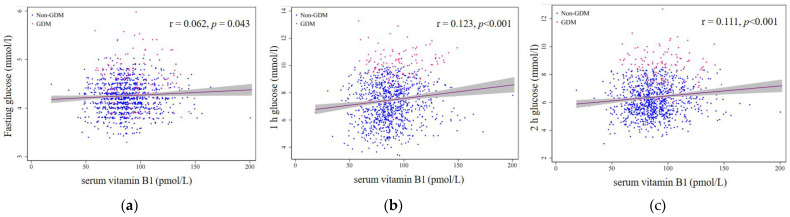
Scatter plot of serum vitamin B_1_ and glucose levels at OGTT: (**a**) fasting glucose; (**b**) 1 h glucose; (**c**) 2 h glucose. The lines indicate the straight-line correlations, and the grey–shaded areas represent 95% confidence intervals (CI).

Table 2 shows the correlations between serum B vitamins and blood glucose levels at OGTT. Serum vitamin B_1_ was positively correlated with fasting, 1 h and 2 h plasma glucose, with Pearson correlation coefficients of 0.062, 0.123 and 0.111, respectively (Figure 1). Moreover, significant positive correlations were found between serum levels of vitamin B_1_ and vitamin B_6_, whereas negative correlations were found between serum levels of folate and vitamin B_12_ (Table 3).

### 3.3. Associations between Serum B Vitamins and GDM Risk

**Figure 2:**
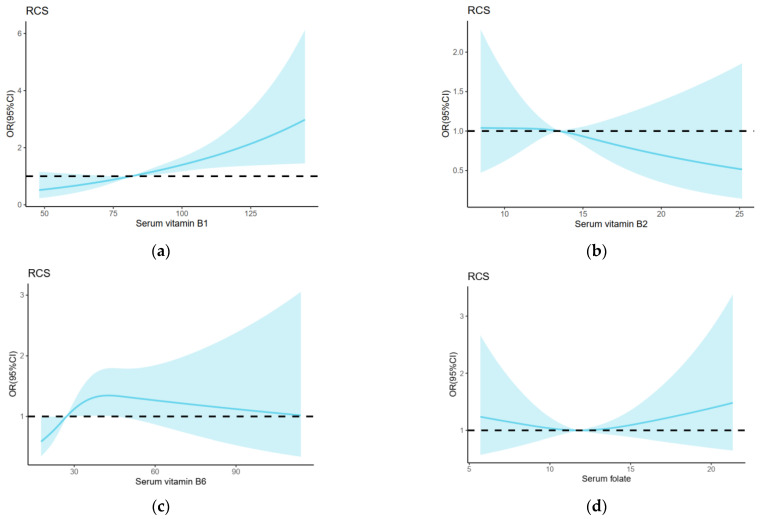

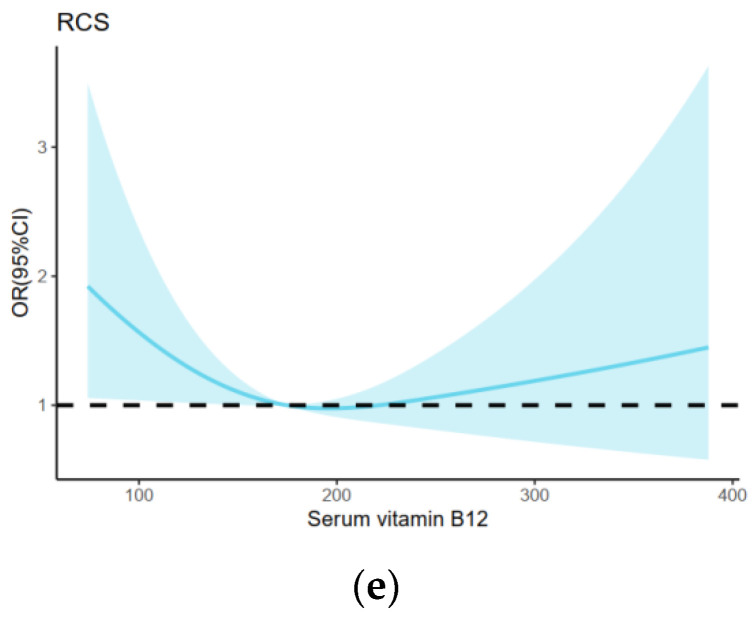
Restricted cubic spline (RCS) regression analysis of group B vitamins including (**a**) serum vitamin B_1_, (**b**) serum vitamin B_2_, (**c**) serum vitamin B_6_, (**d**) serum folate, and (**e**) serum vitamin B_12_ with GDM risk. The median of vitamin B_1_ 86.5 pmol/L, B_2_ 13.5 pmol/L, B_6_ 27.2 pmol/L, folate 11.8 nmol/L and B_12_ 174.8 pmol/L was selected as the reference levels, respectively. The lines indicate estimated ORs, and the light-blue–shaded areas represent 95% CI.

Table 4 shows the adjusted ORs (aOR) and 95% CIs estimated based on the quartiles of serum B vitamins and GDM risks. An obvious positive increased risk trend across vitamin B_1_ quartile groups with GDM risk was observed (*p*-Trend = 0.001). Furthermore, high serum levels of vitamin B_6_ were associated with an increased risk of GDM. Compared with women in the lowest quartile of vitamin B_6_, those in the upper two quartiles had approximately twofold higher odds of GDM (aOR 1.93 [95% CI 1.08–3.43], *p* = 0.026; aOR 1.84 [95% CI 1.03–3.29], *p* = 0.040). However, no obvious increased risk trend with GDM was found across vitamin B_6_ quartile groups (*p*-Trend = 0.054). Compared with vitamin B_12_ levels < 150 pmol/L, levels > 150 pmol/L were associated with a lower risk of GDM (aOR 0.57 [95% CI 0.38–0.84]; *p* = 0.005). No significant associations were found between serum vitamin B_2_, folate levels or the ratio of folate and vitamin B_12_ and GDM risks. RCS regression models revealed that serum vitamin B_6_ (*p* = 0.048) and vitamin B_12_ (*p* = 0.033) were associated with an increased risk of GDM in a nonlinear fashion. No significant associations were found between serum vitamin B_2_ and folate levels and GDM risks in RCS regression models (Figure 2).

## 4. Discussion

In this prospective cohort study, we investigated the association between serum levels of B vitamins in early pregnancy and the incidence of GDM at 24–28 weeks of gestation. We found that the risk of GDM increased in a dose–response manner across serum vitamin B_1_ quartiles one to four during early pregnancy, after comprehensively adjusting for a number of covariables. Consistent with this, positive correlations between serum vitamin B_1_ levels as a continuous variable with plasma fasting, OGTT 1 h and 2 h glucose levels were observed in the present study. Moreover, women in the upper two quartiles of serum vitamin B_6_ levels had higher odds of GDM. In addition, serum vitamin B_12_ levels > 150 pmol/L had a protective effect on GDM incidence. However, the associations between serum folate or the serum folate/vitamin B_12_ and GDM risks were not detected in the present study.

Vitamin B_1_ is an essential micronutrient involved in glucose metabolism in almost all living organisms. The Chinese dietary guidelines recommend a dietary reference intake of 1.2 mg per day for healthy adult women and pregnant women in the first trimester, and 1.4 mg and 1.5 mg per day in the second and third trimesters, respectively [29]. This recommendation reflects increased requirements for energy and carbohydrates during pregnancy. Vitamin B_1_ levels are often reduced in individuals with dietary patterns rich in carbohydrates and in those with diabetic neuropathy. Routine intake of vitamin B_1_ supplements for disease prevention is not recommended during pregnancy.

To date, only a few studies have evaluated the associations between vitamin B_1_ (including food intake or body status) and the risks of diabetes, and inconsistent findings have been reported. Recently, a national prospective study in the Chinese population has revealed a U-shaped association between dietary vitamin B_1_ intake and new-onset diabetes [30]. Furthermore, an ecological study revealed that the increased prevalence of diabetes in an American population was significantly and positively correlated with an increased consumption of vitamin B_1_ [31]. However, Thornalley reported that a low level of plasma vitamin B_1_ was prevalent in diabetes patients [32]. These conflicting findings may result from different study designs and populations, especially cohorts with particular diseases and dietary patterns. Therefore, the association between vitamin B_1_ intake and vitamin B_1_ body status and the risk of diabetes remains uncertain. These findings also imply an important role of population background in determining health consequences. As far as we know, our study provides the first data that a high serum vitamin B_1_ concentration in early pregnancy may bring about a subsequent risk of GDM. However, the exact mechanisms linking optimal vitamin B_1_ intake and serum vitamin B_1_ levels and the risk of GDM are still not clear. More studies are needed to confirm our findings and explore the underlying mechanisms among pregnant women.

Vitamin B_6_ functions as a coenzyme for many of the enzymes involved in the metabolism of glucose, lipids, amino acids, DNA and neurotransmitters [33]. In addition, vitamin B_6_ can quench reactive oxygen species as an antioxidant molecule [34]. It can be found in several foods including fish, meat, nuts and fresh vegetables, with recommendations of 1.4 mg daily for adults and 2.2 mg daily for pregnant women in the Chinese dietary guidelines [29]. In clinical practice, vitamin B_6_ has been used to alleviate nausea and vomiting caused by pregnancy status [35]. Plasma levels of pyridoxal 5-phosphate, an active metabolite of vitamin B_6_, are decreased in conditions with elevated alkaline phosphatase such as liver and bone diseases, diabetes and cancer; therefore, the measurement of total B_6_ (as in our study) has been recommended as a direct marker of B_6_ status in pregnant women [20].

Animal studies have shown that vitamin B_6_ deficiency in pregnancy may increase the risk of glucose intolerance by disturbing the catabolism of tryptophan into serotonin, which is critical for β-cell proliferation during pregnancy [36]. However, one study revealed that in mice with vitamin B_6_ deficiency, insulin levels remained intact, though insulin resistance increased [37]. In addition, vitamin B_6_ administration does not affect blood glucose levels in women with GDM [38]. Our study showed a nonlinear association between serum vitamin B_6_ levels and the risk of GDM, with women in the upper two quartiles having a higher risk of GDM. Of note, we found a positive relationship between vitamin B_1_ and vitamin B_6_ (r = 0.063, *p* < 0.05) and the former was positively correlated with GDM risk as previously mentioned. One possible explanation is that high vitamin B_6_ level was related to elevated appetite, energy intake and body weight. Additional research is required to investigate the underlying mechanisms involved in the relationship between vitamin B_6_ and GDM.

Vitamin B_6_, folate and vitamin B_12_ are of great importance in fetal development because of their role in one-carbon metabolism, which is crucial for the synthesis of DNA, the conversion of homocysteine to methionine, neurological function, and the formation of red blood cells [12,39]. Folate is a key nutrient for pregnant women. Recommendations for synthetic folic acid supplementation in pregnant women and women preparing for pregnancy are part of public health strategies to prevent birth defects [14]. A deficiency of vitamin B_12_ in pregnancy can induce anemia, homocysteinemia, cardiovascular dysfunction, neurological disorders and oxidative stress [40]. Vitamin B_12_ is only present in animal sources; therefore, vegans, vegetarians and pregnant women who suffer from pregnancy-associated nausea and vomiting are at risk of B_12_ deficiency [40]. A recent meta-analysis found that vitamin B_12_ insufficiency was common in pregnant women, with pooled estimates of 21%, 19% and 29% in the first, second and third trimesters, respectively. Furthermore, geographic differences in the maternal prevalence of vitamin B_12_ deficiency were observed, with the highest prevalence reported in India (70–74%) [41].

In our study population, 32.9% (350/1065) of women had vitamin B_12_ insufficiency. Previous evidence suggests that vitamin B_12_ deficiency increases the risk of GDM, which was in line with our findings [42,43]. The relationship between serum B_12_ levels and GDM was nonlinear with our RCS model, and a serum vitamin B_12_ level of ≥150 pmol/L reduced the risk of GDM by 43%. Several mechanisms have been proposed to explain the protective effects of vitamin B_12_ on diabetes; although, none have been proven. Vitamin B_12_ has a negative effect on homocysteine metabolism, and an association exists between hyperhomocysteinemia and insulin resistance; furthermore, oxidative stress is caused by vitamin B_12_ deficiency [44]. In animal studies, low levels of vitamin B_12_ increase lipid accumulation in adipocytes and trigger dyslipidemia, leading to β-cell lipotoxicity [45]. Vitamin B_12_ is a coenzyme involved in the degradation of odd-chain fatty acids and BCAAs [18]. Increased dietary and plasma levels of BCAAs are correlated with obesity, insulin resistance and diabetes [46].

Vitamin B_12_ has a close metabolic inter-relationship with folate. It is required for the conversion of N5-methyl-tetrahydrofolate into tetrahydrofolate, which is the active form of folate involved in the synthesis of DNA and the methionine cycle [12]. The levels of these two biomarkers were significantly and inversely correlated in our population (r = −0.087, *p* < 0.05). The ratio of folic acid/vitamin B_12_ and GDM risk has been investigated in several studies [15,16,17] and analyzed in a meta-analysis [47]. Nevertheless, these studies have yielded contradictory results with negative, positive and no link detected. No association between this ratio in early pregnancy and the risk of GDM was detected in the current study. Possible reasons for the contradictory findings include differences in the following: study design, vitamin supplements used in different populations, and gestational age at the time of sampling (e.g., early pregnancy vs. middle or late pregnancy). Intriguingly, the serum levels of vitamin B_12_ were highest in women who took folic acid supplements in our study (Supplementary Materials Table S1). Further large-scale longitudinal studies and trials on vitamin B_12_ supplementation are necessary to clarify the relationships between folic acid/vitamin B_12_ status and GDM risk. The aim will be to determine the dose of these two vitamins to achieve an optimum balance throughout pregnancy.

Several limitations of this study should be noted. First, we controlled for a number of covariates; however, residual confounding may not be eliminated as we had no information on diet and liver function tests. Second, this study was conducted in Shanghai; therefore, whether the findings observed here can be translated to other populations needs further verification. Third, the lack of pre-specified power calculations for sample size in the study might limit the strength of the evidence regarding the association between B vitamins and GDM.

## 5. Conclusions

This cohort study in China showed that higher maternal serum vitamin B_1_ and vitamin B_6_ levels in early pregnancy are significantly associated with increased GDM risk. In addition, sufficient vitamin B_12_ status is significantly associated with a lower GDM risk. Our findings suggest that the body status of B vitamins in early pregnancy is a potential predictive biomarker of GDM. Further research is necessary to determine the appropriate levels of B vitamins in early pregnancy to optimize maternal and offspring health.

## Figures and Tables

**Table 1:** The basic characteristic of the study population by gestational diabetes mellitus (GDM) status (N = 1065).

Characteristics	All	GDM (*n* = 121)	Non-GDM (*n* = 944)	*p*-Value
Age	30.8 ± 3.7	32.2 ± 3.8	30.6 ± 3.6	<0.001
Education background (*n* (%))				0.381
≤Senior high school	68 (6.4)	12 (9.9)	56 (5.9)	
College	693 (65.1)	76 (62.8)	617 (65.4)	
≥Postgraduate degree	304 (28.5)	33 (27.3)	271 (28.7)	
Smoking exposure (*n* (%))	131 (12.3)	19 (15.7)	112 (11.9)	0.226
Alcohol drinking (*n* (%))	108 (10.1)	9 (7.4)	99 (10.5)	0.296
Pre-conceptional body mass index (kg/m^2^) (*n* (%))				<0.001
<18.5	159 (14.9)	9 (7.4)	150 (15.9)	
18.5–24	726 (68.2)	76 (62.8)	650 (68.9)	
>24	180 (16.9)	36 (29.8)	144 (15.3)	
First-degree family history of diabetes (*n* (%))				<0.001
Yes	136 (12.8)	24 (19.8)	112 (11.9)	
No	906 (85.1)	90 (74.4)	816 (86.4)	
Unclear	23(2.2)	7 (5.8)	16 (1.7)	
Primiparity (*n* (%))	874 (82.1)	91 (75.2)	783 (82.9)	0.037
Gestational weight gain at OGTT visits (kg)	7.9 ± 3.8	8.2 ± 3.6	7.9 ± 3.8	0.478
Physical activity level ≥ 600 MET (*n* (%))	346 (32.5)	45 (37.2)	301(31.9)	0.241
B-vitamin supplements (*n* (%))				0.671
Folate supplements	276 (25.9)	28 (23.1)	248 (26.3)	
Multivitamin supplements	728 (68.4)	87 (71.9)	641 (67.9)	
No	61(5.7)	6 (5.0)	55 (5.8)	
Biochemical characteristics				
B_1_ (pmol/L)	86.5 (75.1–98.5)	95.9 (78.5–110.2)	86.1 (74.7–97.5)	<0.001
B_2_ (pmol/L)	13.5 (12.3–14.9)	13.2 (11.9–14.7)	13.5 (12.3–14.9)	0.406
B_6_ (pmol/L)	27.2 (24.2–35.7)	28.9 (24.8–37.4)	26.9 (24.1–35.4)	0.069
Folate (nmol/L)	11.8 (10.1–13.9)	11.7 (10.3–14.0)	11.8 (10.1–13.9)	0.952
Folate insufficiency at <5.9 nmol/L	14 (1.3)	2 (1.7)	12 (1.3)	0.729
B_12_ (pmol/L)	174.8(132.6–210.3)	160.0 (124.7–194.8)	176.1 (134.5–211.1)	0.038
B_12_ insufficiency at <150 pmol/L	350 (32.9)	54 (44.6)	296 (31.4)	0.003
Homocysteine (umol/L)	6.6 (6.0–7.5)	6.7 (5.9–7.7)	6.6 (6.0–7.5)	0.828

OGTT, oral glucose tolerance test; MET, metabolic equivalent.

**Table 2:** The correlation of serum B vitamins and glucose levels in the cohort study ^1^.

OGTT	Vitamin B_1_	Vitamin B_2_	Vitamin B_6_	Folate	Vitamin B_12_
Fasting	0.062 *	−0.038	0.048	−0.010	0.051
1 h	0.123 *	0.003	0.011	0.025	−0.027
2 h	0.111 *	0.030	0.036	0.012	0.010

^1^ Pearson correlation coefficient was shown, * *p* < 0.05.

**Table 3:** The correlation of various serum B vitamins in the cohort study ^1^.

	Vitamin B_1_	Vitamin B_2_	Vitamin B_6_	Vitamin B_12_	Folate
Vitamin B_1_	1.000				
Vitamin B_2_	−0.008	1.000			
Vitamin B_6_	0.063 *	−0.037	1.000		
Vitamin B_12_	0.038	−0.055	−0.003	1.000	
Folate	−0.052	0.032	−0.022	−0.087 *	1.000

^1^ Pearson correlation coefficient was shown, * *p* < 0.05.

**Table 4:** Association of maternal serum vitamin B_1_, vitamin B_2_, vitamin B_6_, folate, and vitamin B_12_ in early pregnancy with GDM risk (N = 1065).

Variables	GDM/Total (%)	Model 1 ^†^			Model 2 ^‡^		
		OR	95% CI	*p*-Value	OR	95% CI	*p*-Value
Vitamin B_1_							
Q1	23/276 (8.33)		reference			reference	
Q2	21/266 (7.89)	0.91	0.50–1.69	0.763	0.93	0.49–1.77	0.831
Q3	30/266 (11.28)	1.35	0.76–2.39	0.305	1.49	0.82–2.69	0.188
Q4	47/266 (17.67)	2.28	1.34–3.87	0.002	2.20	1.27–3.82	0.005
*p*-Trend				<0.001			
Vitamin B_2_							
Q1	34/268 (12.69)		reference			reference	
Q2	30/266 (11.28)	0.87	0.52–1.48	0.617	0.88	0.51–1.50	0.631
Q3	29/267 (10.86)	0.84	0.49–1.42	0.513	0.91	0.53–1.57	0.738
Q4	28/264 (10.61)	0.82	0.48–1.39	0.455	0.73	0.42–1.28	0.276
*p*-Trend				0.435			0.310
Vitamin B_6_							
Q1	22/267 (8.24)		reference			reference	
Q2	27/266 (10.15)	1.26	0.70–2.27	0.446	1.27	0.69–2.33	0.437
Q3	36/266 (13.53)	1.74	1.00–3.05	0.052	1.93	1.08–3.43	0.026
Q4	36/266 (13.53)	1.74	1.00–3.05	0.052	1.84	1.03–3.29	0.040
*p*-Trend				0.069			0.054
Folate							
Q1	27/267(10.11)		reference			reference	
Q2	38/269 (14.13)	1.46	0.86–2.47	0.156	1.55	0.90–2.68	0.112
Q3	22/263 (8.37)	0.81	0.45–1.46	0.488	0.90	0.49–1.66	0.745
Q4	34/266 (12.78)	1.30	0.76–2.23	0.334	1.41	0.81–2.45	0.225
*p*-Trend				0.697			0.499
Vitamin B_12_							
Q1	37/267(13.86)		reference			reference	
Q2	34/266 (12.78)	0.91	0.55–1.50	0.715	0.90	0.54–1.51	0.697
Q3	26/266 (9.77)	0.67	0.40–1.15	0.146	0.71	0.41–1.23	0.219
Q4	24/266 (9.02)	0.62	0.36–1.06	0.082	0.63	0.36–1.11	0.110
*p*-Trend				0.050			0.079
<150 pmol/L	54/350 (15.43)		reference			reference	
≥150 pmol/L	67/715 (9.37)	0.57	0.39–0.83	0.004	0.57	0.38–0.84	0.005
Folate/B_12_							
Q1	28/267 (10.49)		reference			reference	
Q2	23/266 (8.65)	0.81	0.45–1.44	0.471	0.81	0.45–1.47	0.497
Q3	35/265 (13.21)	1.30	0.77–2.20	0.332	1.23	0.71–2.12	0.464
Q4	35/267 (13.11)	1.29	0.76–2.19	0.349	1.37	0.79–2.36	0.258
*p*-Trend				0.176			0.121

^†^ Univariate model. ^‡^ Adjusted for age, education, parity, first-degree family history of diabetes, smoking exposure, alcohol drinking, pre-conceptional body mass index, gestational weight gain at OGTT visits and physical activity levels. OR, odds ratio; Q, quartile.

## Data Availability

The data used in this study can be acquired on request from the corresponding author.

## Supplementary Materials

The following supporting information can be downloaded at: https://www.mdpi.com/article/10.3390/nu14235016/s1, Table S1: Serum levels of B vitamins according to B-vitamin supplements intake in the cohort.
