# Supplementary material for: The Association between Maternal B Vitamins in Early Pregnancy and Gestational Diabetes Mellitus: A Prospective Cohort Study
Source: Nutrients. 2022 Nov 25;14(23):5016. doi: 10.3390/nu14235016 (PMC9736031; doi:10.3390/nu14235016)
Supplement: Supplementary file 1 [file nutrients-14-05016-s001.zip › nutrients-2024367-Supplementary materials.pdf]

Supplementary materials

Table S1. Serum levels of B vitamins according to B-vitamin supplements intake in the cohort[1].

Serum B vitamins	No supplements (n=61)	Multivitamin supplements (n=728)	Folate supplements (n=276)	*p*-Value
B_1(pmol/l)	86.3 (80.3-96.4)	86.7 (75.8-98.6)	86.0 (74.1-98.8)	0.671
B_2 (pmol/l)	14.1 (13.0-15.2)	13.4 (12.3-14.8)	13.3 (11.9-14.9)	0.013
B_6 (pmol/l)	28.4 (24.0-35.7)	27.3 (24.2-35.4)	26.8 (23.9-35.9)	0.822
Folate (nmol/l)	11.0 (9.5-12.7)	11.9 (10.2-14.1)	11.7 (10.0-13.7)	0.021
B_{12} (pmol/l)	177.0 (143.8-210.8)	171.5 (128.2-206.7)	187.0 (146.1-217.4)	0.005

[1] Kruskal – Wallis tests were performed.
